# VOCs Are Relevant Biomarkers of Elicitor-Induced Defences in Grapevine

**DOI:** 10.3390/molecules26144258

**Published:** 2021-07-13

**Authors:** Christelle Lemaitre-Guillier, Christelle Dufresne, Agnès Chartier, Stéphanie Cluzet, Josep Valls, Lucile Jacquens, Antonin Douillet, Nicolas Aveline, Marielle Adrian, Xavier Daire

**Affiliations:** 1 Agroécologie, AgroSup Dijon, CNRS, INRAE, Univ. Bourgogne, Univ. Bourgogne Franche-Comté, 21000 Dijon, France; Lucile.Jacquens@u-bourgogne.fr (L.J.); Marielle.Adrian@u-bourgogne.fr (M.A.); xavier.daire@inrae.fr (X.D.); 2 Institut de Chimie Organique et Analytique, ICOA, UMR 7311, Université d’Orléans, rue de Chartres, BP 6759, CEDEX 2, 45067 Orléans, France; christelle.dufresne@univ-orleans.fr (C.D.); agnes.chartier@univ-orleans.fr (A.C.); 3 Equipe Molécules d’Intérêt Biologique, ISVV, Unité de Recherche Œnologie, EA 4577, USC 1366 INRAE, Faculté des Sciences Pharmaceutiques, Université de Bordeaux, CEDEX, 33882 Villenave d’Orno, France; stephanie.cluzet@u-bordeaux.fr (S.C.); josep.valls-fonayet@u-bordeaux.fr (J.V.); 4 Institut Français de la Vigne et du Vin (IFV), 33290 Blanquefort, France; Antonin.DOUILLET@vignevin.com (A.D.); Nicolas.AVELINE@vignevin.com (N.A.)

**Keywords:** grapevine, elicitor, plant defence, volatile organic compounds, terpenes, stir bar sorptive extraction SBSE-GC-MS

## Abstract

Grapevine is susceptible to fungal diseases generally controlled by numerous chemical fungicides. Elicitors of plant defence are a way of reducing the use of these chemicals, but still provide inconsistent efficiency. Easy-to-analyse markers of grapevine responses to elicitors are needed to determine the best conditions for their efficiency and position them in protection strategies. We previously reported that the elicitor sulphated laminarin induced the emission of volatile organic compounds (VOCs) by grapevine leaves. The present study was conducted to characterise and compare VOC emissions in response to other elicitors. Bastid^®^ was first used to test the conditions of VOC collection and analysis. Using SBSE-GC-MS, we detected several VOCs, including the sesquiterpene α-farnesene, in a time-dependent manner. This was correlated with the induction of farnesene synthase gene expression, in parallel with stilbene synthesis (another defence response), and associated to resistance against downy mildew. The other elicitors (Redeli^®^, Romeo^®^, Bion^®^, chitosan, and an oligogalacturonide) induced VOC emission, but with qualitative and quantitative differences. VOC emission thus constitutes a response of grapevine to elicitors of various chemical structures. Therefore, VOC analysis is relevant for studying the impact of environmental factors on grapevine defence responses and optimising the performance of elicitors in vineyards.

## 1. Introduction

Grapevine (*Vitis vinifera* L.) is an economically important crop highly susceptible to fungal diseases such as downy mildew and powdery mildew, caused by the oomycete *Plasmopara viticola* (*Pv*) and the ascomycete *Erysiphe necator*, respectively. Controlling these diseases mainly relies on the application of chemical fungicides. However, the extensive use of these pesticides raises environmental and health issues. Elicitor-induced resistance (IR) of plants against diseases is an attractive strategy for reducing the use of chemical fungicides. Elicitors trigger plant defence reactions, resulting in antimicrobial production or/and cell-wall reinforcement that stop or limit pathogen infection [1]. Elicitors are compounds of various biochemical classes, including lipids, proteins, and carbohydrates. Some of them are microbe- or damage-associated molecular patterns (MAMPs and DAMPs), recognised by specific plant cell receptors [2,3]. Other elicitors are phytohormones e.g., jasmonic acid and salicylic acid and some of their chemical functional analogues [4,5].

Successful applications of elicitors in field conditions have been reported [6,7]. Some are commercialised in France to protect grapevine against downy mildew, powdery mildew, and grey mould. For example, elicitors composed of chito-oligosaccharides (COS-OGA), disodium phosphonate or cerevisiane (yeast extract) are commercialised on the French market. Although they are increasingly used, they are not commonly used yet, mainly due to a variable level of efficiency. The plant responsiveness to elicitor application can be influenced by a number of factors such as the plant developmental stage, genotype, abiotic stress, or nutrition [8,9]. Therefore, further investigations are required to optimise this strategy in field conditions. In this context, relevant, easy-to-analyse biomarkers are needed to monitor the plant defence responses induced by elicitor application, so as to determine the conditions for optimal efficiency of such treatments and in turn their positioning in integrated crop protection strategies. Plant defence compounds are logical candidate markers. Stilbene phytoalexins have been the most studied defence response of grapevine so far [10]. They are antimicrobial compounds inducible by various elicitors such as chitosan [11], methyl jasmonate [4], laminarin [12] or sulphated laminarin [13], and are associated to induced resistance against pathogens [10]. However, their analysis requires plant organ sampling and extraction steps, and is therefore tricky to implement in field studies. Besides stilbenes, volatile organic compounds (VOCs) are secondary metabolites that play important roles in the adaptation of the plant to its environment, e.g., pollination or resistance to abiotic and biotic stresses [14]. Regarding plant–pathogen interactions, VOCs can act indirectly as airborne signals that prime defence reactions against pathogens in neighbouring plants [15,16,17], or directly as antimicrobial compounds [18]. A limited number of works have reported that elicitors can trigger VOC emission in plants [16,19,20,21]. Sulphated laminarin induces VOC emission—mainly mono- and sesquiterpenes—in grapevine [21].

An advantage of VOC analysis is that VOC collection does not require organ sampling, and there is no extraction step. Thus, VOCs may represent valuable, relatively easy to assess markers of grapevine response to elicitors. In order to verify this hypothesis and pave the way for future experiments in field conditions, we analysed and compared VOC emission by grapevine leaves treated by different elicitors, with a special focus on three commercial ones: Bastid^®^ (COS-OGA), Redeli^®^ (disodium phosphonate), and Romeo^®^ (cerevisiane). 

## 2. Results and Discussion

### 2.1. VOC Sampling Method

We first focused on the method of VOC collection with a view to transferring it to the vineyard in the future. In previous studies [21], VOCs were collected from vines individually enclosed in bags using fibres coated with divinylbenzene (DVB), carboxen (CAR), and polydimethylsiloxane (PDMS). Solid-phase microextraction (SPME) has proved to be a useful tool to collect plant VOCs [22]. It uses a fused silica fibre coated with a polymeric material to adsorb and pre-concentrate volatile compounds. However, SPME lacks sensitivity because of the low amount of polymeric sorbent available on fibre. Moreover, fibres are fragile and can break easily, so they are difficult to use in the field. Therefore, we also assessed Twister^TM^, another sensor of the Stir Bar Sorptive Extraction (SBSE) type. Twisters^TM^ are magnetic stir rods coated with a polymeric material. They have a more significant sorbent volume than SPME fibre has. Like SPME fibres, SBSE can be used to pre-concentrate and adsorb volatile compounds in gas samples. In the present study, an SPME fibre and a stir bar were both placed in the foliage of a same plant in order to compare the nature and the intensity of the collected VOCs. The experiment was performed using six plants individually wrapped in a bag and elicited with sulphated laminarin (PS3, 2.5 g/L), as previously described by Chalal, et al. [21]. VOCs were collected 3 days (D3) after elicitation.

**Figure 1:**
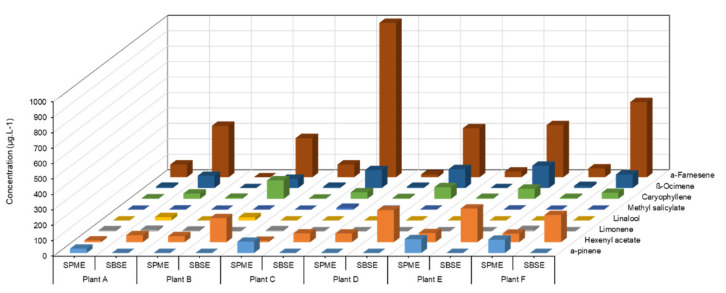
Comparison of VOC signal responses from elicitor-treated grapevine leaves, detected by SPME fibre and SBSE stir bar in bag containment. Amounts (µg/L) of eight targeted VOCs collected for 4 h on D3 after sulphated laminarin (PS3, 2.5 g/L) treatment of four plants (A to D) individually placed in a bag, using SPME fibres and SBSE stir bars (Twisters^TM^) as sensors.

The concentration (µg/L) of detected volatiles was determined using external calibration with monoterpene, sesquiterpene, and green leaf volatile (GLV). These compounds were identified against the NIST-14 library and the retention times of the corresponding standards. As previously reported, PS3 induced VOC emission in grapevine [21]. Targeted analysis of eight VOCS showed that SBSE led to a better signal response for hexenyl acetate, β-caryophyllene, β-ocimene, and α-farnesene, while α-pinene was better detected by SPME (Figure 1). SPME fibre lacks sensitivity for oxygenated and linear terpenes (linalool, β-ocimene), sesquiterpenes (β-caryophyllene, α-farnesene) and GLVs (hexenyl acetate, methyl salicylate). Therefore, SBSE stir bars, even coated with a non-polar adsorbent (PDMS), could be considered a more appropriate sensor for the sampling of VOCs of large polarity and chemical nature.

**Figure 2:**
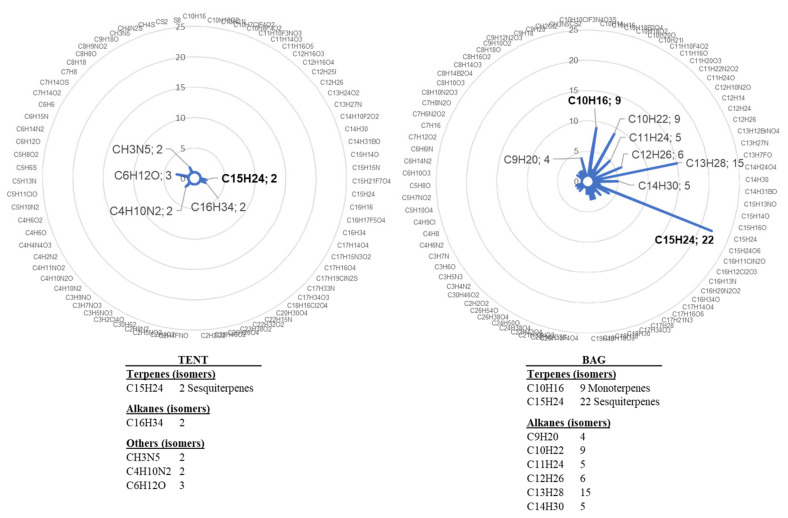
Chemical formulas detected in the bag or the tent containments. VOCs were collected for 4 h from four plants treated with water and individually enclosed in bags (bag) and from six plants gathered in a tent (tent). Radar plot illustrations correspond to the combined lists of VOCs detected on D3 and D5 after treatment. Chemical formulas were determined by GC-MS analysis and NIST identification.

We also assessed another mode of bagging plants for VOC trapping. In previous experiments [21], grapevines were individually enclosed in bags. However, the inner side of the bag was quickly covered with mist due to plant transpiration. The level of the analysed VOC was also quite variable, maybe due to physical stress related to the bag and/or variability of the response among plants (Figure 1). For these reasons, we analysed VOC emission by plants grouped under a tent and compared it with that of plants enclosed individually, following water treatment. Sampling was performed on day 1 (D1), day 3 (D3), and day 5 (D5) after water treatment. The comparison of VOC emission showed huge differences between the two collection systems, supporting our hypothesis of a stress related to individual bagging. We indeed observed a higher number and diversity of water-induced VOCs in the bag containment mode (Figure 2). In view of these results, the tent mode was retained for the next experiments because it appeared less stressful for grapevine. 

### 2.2. SBSE-GC-MS Analysis of VOC Emission Elicited by Bastid^®^

We first assessed VOC production induced by the marketed Bastid^®^ elicitor. Bastid^®^ is a mixture of chitooligosaccharides (COS) and oligogalacturonides (OGA) as active components, in which OGA dimers formed in the presence of calcium ions are stabilised by COS. This complex exhibits higher eliciting activity in Arabidopsis cell suspensions than the individual components do [23]. It triggers plant defence in tomato [24] and potato [25], and controls diseases of a number of crops. It is marketed for control of grape powdery mildew and downy mildew.

In order to assess a basal blend of the experimental system (containment atmosphere + Bastid^®^), preliminary analyses of the VOCs collected in a tent where Bastid^®^ or water were sprayed in the absence of plants were performed (data not shown). The molecules detected following water treatment were 2-aziridinylethyl amine; acetic acid, methyl ester; cyclic octaatomic sulphur; oxime-, methoxy-phenyl-, and toluene. The molecules detected following Bastid^®^ treatment were glycidol; toluene; 2-propenoic acid, 3-(3,4-dimethoxyphenyl)-, (*E*)-fumaronitrile, and nonane, 2,2,4,4,6,8,8-heptamethyl-. Toluene was detected following both treatments; it may have been present in the ambient atmosphere of the greenhouse. Others may have come from water contaminants, Bastid^®^ degradation products, and pollutants such as sprayer components.

Next, we analysed the VOCs emitted by Bastid^®^-treated plants, collected on D1, D3, and D5. PCA analysis of the data showed time-dependent VOC emission (Figure S1). The data obtained from the scan mode analysis of the samples collected at the three time points from three independent experiments were filtered according to ANOVA significance and to the fold change (FC) relative to H_2_O control criteria (significant for *p* < 0.05, induced above 1.5-fold at least twice in the three experiments). As a result, seven VOCs were selected. Two of them were sesquiterpenes: α-farnesene and β-caryophyllene (Table 1). The other ones were decanal; toluene; 2-oxo-4-phenyl-6-(4-chlorophenyl)-1,2-dihydropyrimidine; 4,4′-bi-4*H*-pyran, 2,2′,6,6′-tetrakis(1,1-dimethylethyl)-4,4′-dimethyl- and 3,4-dihydroisoquinolin-7-ol, 1-[4-hydroxybenzyl]-6-methoxy-. α-farnesene, β-caryophyllene, decanal, and toluene can be produced and emitted by plants [26,27]. However, 2-oxo-4-phenyl-6-(4-chlorophenyl)-1,2-dihydropyrimidine; 4,4′-bi-4*H*-pyran, 2,2′,6,6′-tetrakis(1,1-dimethylethyl)-4,4′-dimethyl- and 3,4-dihydroisoquinolin-7-ol, 1-[4-hydroxybenzyl]-6-methoxy- probably did not originate from the plants despite their significant accumulation after elicitor treatment. It is unlikely that these compounds were present in the elicitor formulation as they were not systematically detected following Bastid^®^ treatment (Table 1), or in the plant-less tents sprayed with Bastid^®^. Their origin remains unexplained. 

The release of these VOCs was time-dependent, with a variable time of significant emission depending on the VOC and the experiment (Table 1). The profile of significant VOC emission in response to Bastid^®^ was also variable among experiments (Table 1). For example, three of them (toluene; 4,4′-bi-4*H*-pyran, 2,2′,6,6′-tetrakis(1,1-dimethylethyl)-4,4′-dimethyl- and 3,4-dihydroisoquinolin-7-ol, 1-[4-hydroxybenzyl]-6-methoxy-) were not detected in experiment 2. The sesquiterpene α-farnesene was the only VOC observed in all three experiments, with FC > 1.5 at two collection times (D3 and D5). It was also accumulated at ratios among the highest ones (Table 1). α-farnesene production had already been detected in response to sulphated laminarin Chalal, et al. [21]. Its emission had also been reported in different plant/pathogen interactions [28]. To complete the study of α-farnesene induction by Bastid^®^, we monitored the expression of the *VvFAR* gene, coding for the grapevine (*E,E*)-α-farnesene synthase family. *VvFAR* was up-regulated as early as D1 (transcripts accumulated 7- to 14-fold compared to the water control) and was still highly induced on D4 (14 to 28-fold compared to water) (data not shown).

Bastid^®^ systematically induced VOC emission by grapevine leaves in our experimental conditions, although the time course of emission, the nature, and the quantity of the significantly emitted compounds varied. α-farnesene was highlighted as systematically induced by Bastid^®^.

### 2.3. Comparison of VOC Emission Elicited by Other Elicitors

In addition to Bastid^®^, two other commercial elicitors—Redeli^®^ and Romeo^®^—were tested on grapevine plants. VOC analysis was performed, and the results were compared. Redeli^®^ is a phosphonate-based product with anti-oomycete and elicitor activities, used to control grapevine downy mildew [29,30]. Romeo^®^ is made up of cerevisiane extracted from yeast cell walls [31] and is marketed to control grapevine diseases like downy mildew, powdery mildew, and grey mould.

**Figure 3:**
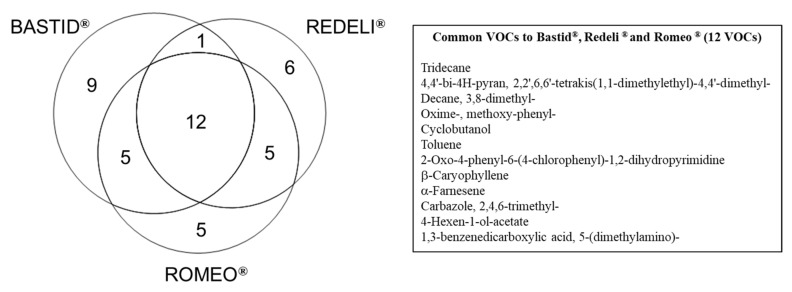
Venn diagram of the VOCs induced by commercial elicitors. Distribution of 43 VOCs detected with FC > 1.5 at least once at two time points or at least in two replicates for one time point (Table 2), following Bastid^®^, Redeli^®^ or Romeo^®^ treatment. The 12 commonly induced VOCs are listed in the table on the right.

For each elicitor, time point, and replicate, the data obtained from the scan mode analysis were filtered according to the fold change (FC > 1.5) relative to the H_2_O control. This procedure provided a list of 43 VOCs induced at least by one elicitor and in two replicates of the experiment at one time point/in one replicate at two time points (Table 2). Among them, 23 fitted these criteria for the three elicitors (Table 2 and Figure 3): 4,4′-bi-4*H*-pyran, 2,2′,6,6′-tetrakis(1,1-dimethylethyl)-4,4′-dimethyl-; decane, 3,8-dimethyl-; oxime-, methoxy-phenyl-; cyclobutanol; toluene; 2-oxo-4-phenyl-6-(4-chlorophenyl)-1,2-dihydropyrimidine; β-caryophyllene; α-farnesene; carbazole, 2,4,6-trimethyl-; 4-hexen-1-ol-acetate; 1,3-benzenedicarboxylic acid, 5-(dimethylamino)-; borane, diethyl(decyloxy)-; dodecane; sulphurous acid, 2-ethylhexyl hexyl ester; benzene, (1-ethyldecyl)-; undecane, 3,5-dimethyl-; benzene, (1-butyloctyl)-; decane, 2,3,8-trimethyl-; d-limonene; benzaldehyde; 4-chlorobenzoic acid, 4-nitrophenyl ester and succinic acid, 2,4,6-trichlorophenyl 2-naphthylmethyl ester. Among those, 12 commonly induced VOCs (listed in Figure 3) constitute a VOC blend marker of the grapevine response to elicitor treatment. Toluene may have been present in the greenhouse atmosphere, as mentioned above.

As indicated in Figure 3, nine VOCs were specifically induced in response to Bastid^®^ (nonanal; nonane, 2,2,4,4,6,8,8-heptamethyl-; 3-pentanamine; 2,5-cyclohexadiene; 1,4-diethyl-1,4-dimethyl-; *n*-hexadecanoic acid; decanal; methyl methacrylate; 3,4-dihydroisoquinolin-7-ol, 1-[4-hydroxybenzyl]-6-methoxy-; α-pinene); 6 in response to Redeli^®^ (undecane, 2,6-dimethyl-; pyrimidine-2,4(1*H*,3*H*)-dione, 5-amino-6-nitroso-; *cis*-muurola-4(14),5-diene; 3-ethyl-3-methylheptane; butanoic acid, 2-methyl-; 1,2-dimethylpropyl ester, and 3-benzoyl-2-t-butyl-4-isopropyloxazolidin-5-one); and 5 in response to Romeo^®^ (1,2-benzenedicarboxylic acid; bis(2-methylpropyl) ester; 4-ethylbenzoic acid, 2-formyl-4,6-dichlorophenyl ester; methenamine; tetradecane, and methyl salicylate). However, we cannot conclude that they are markers of the grapevine response to the corresponding elicitor because they were not induced (FC > 1.5) in the three replicates of the experiment.

All three elicitors induced VOC emission, although with differences in the number and nature of the VOCs. The time course of VOC emission was also variable. Elicitor-induced VOC release has been reported in a limited number of studies [16,19,20], but to our knowledge a comparative study like ours has never been performed. Moreover, the capacity of a yeast extract and of a phosphonate to enhance grapevine VOC emission is demonstrated for the first time. 

In order to complete this study, VOCs were analysed in response to chitosan and an oligogalacturonide (i.e., COS-OGA, made up of such compounds), as well as Bion^®^. Chitosan and oligogalacturonides are known elicitors of grapevine defence reactions [11,32,33,34]. Their activity depends on their degree of polymerisation and degree of deacetylation for chitosan [11,32,35]. Bion^®^ is also a well-known elicitor of defence reactions whose active ingredient is acibenzolar-S-methyl or benzothiadiazole (BTH), a hormone-like analogue of salicylic acid. BTH has been reported as an elicitor of grapevine defence reactions [29]. These three elicitors also induced VOC emission with different profiles (Table S1). However, a lower number of VOCs was detected following chitosan treatment, and to a lesser extent following Bion^®^ treatment. Few reports have addressed the effect of chitosan on VOC emission by plants. It can induce the release of various VOCs, including mono- and sesquiterpenes in tomato [19] and in rice [20]. Bion^®^ was previously shown to induce VOC emission in bean, and this property was associated with enhanced resistance to a bacterial pathogen [36]. Four VOCs have been recurrently induced by the three elicitors, namely α-farnesene, β-caryophyllene, 2-oxo-4-phenyl-6-(4-chlorophenyl)-1,2-dihydropyrimidine, and toluene. The presence of 2-oxo-4-phenyl-6-(4-chlorophenyl)-1,2-dihydropyrimidine, induced by an elicitor prepared in water (no formulation), rules out the hypothesis of its presence in commercial mixtures. The molecule oxime-, methoxy-phenyl- was detected in the water blank experiment in the absence of plants (data not shown), suggesting that it was present in the greenhouse ambient air. This VOC is listed among the molecules increased in fermentation processes of fish and wine [37,38] or found naturally in bamboo shoots [39], *Pseudomonas aeruginosa* [40], and myxobacteria [41]. As for 1,3-benzenedicarboxylic acid, 5-(dimethylamino)-, its presence is hardly reported. 

VOC emission is therefore consistently induced by elicitors, with different blends despite a relevant core of molecules including α-farnesene. It is now well established that VOCs belong to the defence repertoire of plants against pathogens, especially pests such as caterpillars. In Arabidopsis, β-caryophyllene and GLVs also directly inhibit bacterial growth [42]. The release of GLV 4-hexen-ol-acetate was triggered by insect infestation on maple [27]. Together with oxime-, methoxyphenyl- production, 4-hexen-ol-acetate production can be induced by aphid attacks on apple [26]. VOCs can prime defence reactions like airborne signals [15,16,17] or act directly on targets thanks to their antimicrobial properties [42]. In grapevine, increased VOC emission has been reported in the course of interactions with insects. For instance, the feeding of the Japanese beetle on leaves of a *Vitis labrusca* cv. resulted in higher abundance of the GLV MeSA and terpenes, including β-ocimene and α-farnesene [43]. Moreover, the two latter VOCs were the only ones significantly increased following feeding and oviposition of a xylem-feeding sharpshooter on Chardonnay cultivar [44]. Three recent reports also support the role of VOCs in grapevine defence against *P. viticola*. Resistant cultivars released more mono- and sesquiterpenes than a susceptible one did following *P. viticola* inoculation [45]. Then, higher emission of more than 50 VOCs, including mono- and sesquiterpenes, was reported in similar experiments with resistant cultivars [18]. Finally, the role of VOCs in the resistance of the Georgian cultivar Mgaloblishvili and the hybrid Bianca to downy mildew was recently confirmed and suggested antisporulant activity of four terpenes (farnesene, nenolidol, ocimene, and valencene) [45]. Therefore, treating susceptible cultivars with elicitors could mimic the VOC response of resistant cultivars or hybrids to *P. viticola* infection.

### 2.4. Elicitor-Induced Resistance Against Grapevine Downy Mildew

**Figure 4:**
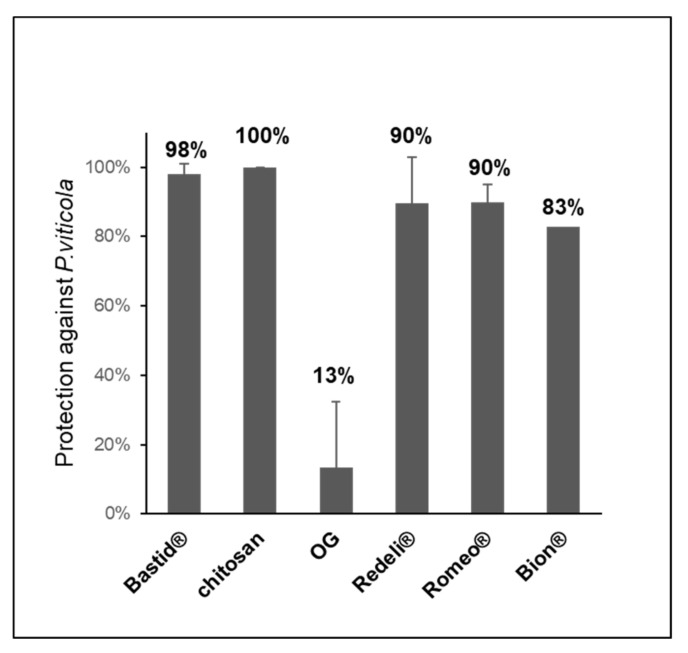
Induced resistance assays. Six elicitors were tested for their efficiency in protecting grapevine against downy mildew (*P. viticola*). Bastid^®^ (0.8 mL/L, 4 assays), Romeo^®^ (2 g/L, 2 assays), Redeli^®^ (1 g/L, 2 assays), OG (2.5 g/L, 2 assays), and chitosan (1 g/L) and Bion^®^ (0.5 g/L, 1 assay). Protection levels (%) were calculated based on comparisons of the sporulating leaf area with the H_2_O control considered as 0% protection.

In parallel to VOC analyses, we assessed the efficiency of the elicitors in the induction of leaf resistance against *P. viticola*. All of them reduced the disease (Figure 4). The highest levels of protection were obtained with chitosan (100%) and Bastid^®^ (98%), followed by Romeo^®^ (89.9%) and Redeli^®^ (89.5%). The oligogalacturonide (OG) turned out to induce the lowest resistance, probably because it has no direct antimicrobial effect; moreover, it was diluted in water in the absence of a surfactant to promote its penetration into the leaves, and in turn elicitor activity [46]. 

### 2.5. Analysis of Stilbenes in Bastid^®^- and Chitosan-Treated Grapevine Plants

**Figure 5:**
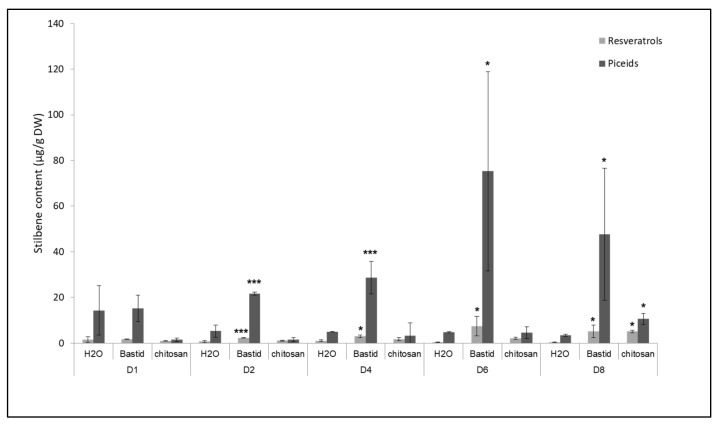
Time course of stilbene production in grapevine leaves treated by Bastid^®^ or chitosan. Treated leaves were collected on D1, D2, D4, D6, and D8 after Bastid^®^, chitosan, and H_2_O (control) treatments. Resveratrol (*trans-* and *cis*-isomers) and piceid (*trans-* and *cis*-isomers) are expressed in µg/g dry weight (DW). Values represent the means ± SD of duplicate experiments. Asterisks (*) indicate significant differences (*p:* *** < 0.001; * < 0.05) between the elicitors and the H_2_O control.

Stilbene phytoalexins were analysed as a reference, as markers of defence elicitation and resistance to diseases [47]. The test was performed with Bastid^®^, used as a reference elicitor in this study, and also with chitosan because it showed the highest level of protection against downy mildew (Figure 4). Four stilbenes, i.e., piceid (*trans*- and *cis-* isomers) and resveratrol (*trans*- and *cis*- isomers) were analysed. Both elicitors induced the accumulation of these compounds (Figure 5). In the water control, the concentrations of resveratrol (0.39 to 1.60 µg/g DW) and piceid (3.40 to 14.38 µg/g DW) were low throughout the 8 days of the experiment compared to those measured after the two elicitor treatments (up to 7.39 and 75.29 µg/g DW for the resveratrol and piceid isomers, respectively). After chitosan treatment, the piceid and resveratrol isomers were only significantly accumulated on D8-3- and 13-fold more than in the H_2_O control, respectively. Bastid^®^ promoted the accumulation of both stilbenes from D2 till D8. It induced higher levels of stilbenes than chitosan did, particularly on D6. Both elicitors triggered the accumulation of phytoalexins. Bastid^®^ induced earlier and higher phytoalexin accumulation than chitosan did, and also higher VOC emission. This observation suggests that the protection of grapevine against downy mildew provided by Bastid^®^ and chitosan involves different modes of action. For Bastid^®^, the induction of phytoalexin synthesis probably plays a key role. Chitosan has long been studied as an elicitor of plant defence [48]. It is very effective to reduce grapevine downy mildew severity in controlled conditions probably thanks to both its direct antimicrobial and indirect elicitor effects. The present study shows that it also induces the emission of terpenes. It may be tempting to conclude that chitosan is a less effective VOC inducer than Bastid^®^ (Table S1). However, it should be noted that the tent experiment was only performed twice. 

## 3. Conclusions

This study shows that VOC emission can be considered as a universal response of grapevine to elicitors of plant defence, even though no specific marker can as yet be highlighted. The elicitors assessed in the present study induced the emission of a common set of VOCs encompassing a dozen chemically different compounds, including the sesquiterpenes α-farnesene and β-caryophyllene. Such a response is concomitant with the induction of stilbene phytoalexins; however, it cannot be directly linked to the level of protection against downy mildew. It rather suggests a combined action of several responses to build an effective defence system against pathogens. Those first results open onto prospects for VOC analysis in field experiments. This could be relatively tricky because of the atmospheric dilution effect and the impact of multiple factors (i.e., plant development and climate) likely to occur, but would also help to identify the critical factors that influence elicitor-induced defence. Such information will be crucial to determine the optimal conditions for efficient elicitor application. Compared to the analysis of other defence responses, VOC analysis has the advantage of being a non-destructive method that could be applied in situ (portable GC-MS apparatus). VOC measurements in the field will also be of interest for chemical ecologists and plant biologists. VOC monitoring indeed contributes to evaluate the impact of environmental stresses and could be useful for improving crop productivity and sustainable defence strategies.

## 4. Materials and Methods

### 4.1. Plant Material 

*Vitis vinifera* L. cv. Marselan (Cabernet sauvignon × Grenache) plants were obtained from herbaceous cuttings placed in individual pots (8 × 8 × 8 cm) containing a mixture of blond peat and perlite (3/1, *v/v*). They were grown in a greenhouse at 24 °C (day)/18 °C (night) with a 16-h photoperiod and at a relative humidity of 70 ± 10% until they developed six leaves (*ca*. 2 months old). They were watered with a fertilising solution (0.25% Topfert2 solution NPK 10-10-10 + oligonutrients, Plantin, France).

### 4.2. Elicitor Treatments

**Figure 6:**
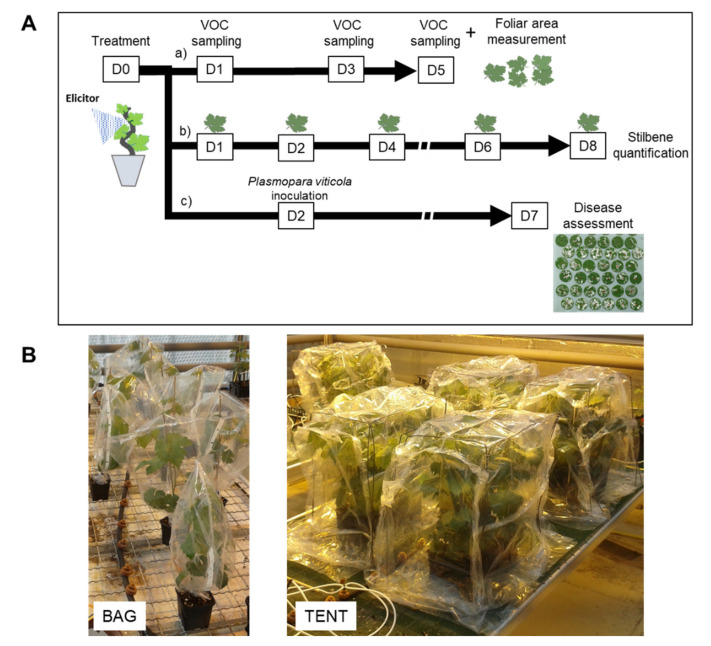
Experimental design. (**A**) Tests on treated grapevine plants. Elicitors or water (control) were sprayed onto the whole plants, at time D0, and then the plants were separated into three sets for further analysis. a) A first set of plants was placed into containments 1, 3, and 5 days after treatment (D1, D3, D5) for VOC sampling. At each of these time points, stir bars were installed in the foliage for 4 h. At the end of the experiment (D5), leaves were picked up to measure leaf area. b) A second set of plants served for stilbene quantification; leaves were detached on D1, D2, D4, D6, and D8. c) A third set was used to assess elicitor-induced resistance against downy mildew. The lower side of the leaves was inoculated with a suspension of *Plasmopara viticola* (*Pv*) sporangia on D2, and the sporulating leaf area was measured on D7. (**B**) Photographs of containment devices used for VOC collection. Plants were either bagged individually (1 plant/bag) or gathered under a sealed tent (six plants/tent) in the presence of VOC sensors.

Six elicitors were used: Bastid^®^, an oligogalacturonide (OG), chitosan, Redeli^®^, Romeo^®^, and Bion^®^ (Table 3). On day 0 (D0), the elicitor solutions were applied as a foliar spray onto the whole plants using a hand-held sprayer (Figure 6A). The products and the doses are detailed in Table 3. Ultrapure H_2_O treatment was systematically used as a negative control in each experiment. The experiments were replicated once for Bion^®^; twice for chitosan; three times for Romeo^®^, Redeli^®^, and OG; and five times for Bastid^®^. 

### 4.3. Induced Resistance Assays

Plants were inoculated with *Pv* 2 days post treatment (dpt) (D2) by spraying a suspension of sporangia at a concentration of 10^4^/mL onto the lower leaf surfaces, as previously described by Trouvelot, et al. [49] (Figure 4). Five days after inoculation (D7), foliar disks were punched out from two leaves of the treated plants and transferred onto humid Whatman paper in translucid Perspex boxes to induce sporulation. The percentage of sporulating area of each disc was determined using image analysis (Visilog), as previously described by Kim Khiook, et al. [50]. Elicitor efficacy was calculated by comparing the sporulating area obtained after treatment *versus* the H_2_O control considered as 0% of protection efficacy.

### 4.4. VOC Collection and Analysis

#### 4.4.1. Sensors

Two types of sensors were tested: *i*—fibres coated with divinylbenzene (DVB), carboxen (CAR), and polydimethylsiloxane (PDMS); StableFlex 1 cm–50/30 μm, (Supelco Inc., Bellefonte, PA, USA), and *ii*—2-cm-long Stir Bar Sorptive Extraction (SBSE) (Gerstel GmbH, Co.KG, Mülheim an der Ruhr, Germany) coated with 1-mm-thick polydimethylsiloxane (PDMS). The stability of the collected volatiles on the stir bar was previously tested under various transportation temperatures and storage conditions until analysis (data not shown). Finally, stir bars were stored at room temperature, sent for analysis 2 weeks after VOC collection at most, and immediately analysed by GC-MS. Before sampling, each stir bar was conditioned in the injector of a gas chromatographer under a stream of helium according to the manufacturer’s recommendations.

#### 4.4.2. Containments

Two types of containments were assessed for VOC collection: i—with plants enclosed individually in a commercial Teflon^®^ oven bag (Albal^®^, Cofresco Frischhalteprodukte GmbH, Co.KG, Minden, Germany), or *ii*—grouped in a tent built with the same material on all six cube faces, as illustrated in Figure 6B. When the individual bag setup was employed, each plant was placed in the bag together with a single stir bar, and four plants were used *per* condition. In the tent device, six plants under the same treatment were gathered with two or three stir bars. The stir bar was magnetically held within the upper part of the foliage using a metal paper clip. The plants were bagged or placed under a tent together with the stir bars for 4 h, from 10:00 a.m. to 2:00 p.m., 1, 3, and 5 dpt (from D1 to D5) (Figure 6A). At the end of the experiment, the leaf areas were measured with an area meter (Li-Cor 3100C) to normalise VOC abundance measured by GC-MS with the leaf emission surface. 

The blank experiment using water and Bastid^®^ sprayed on aluminium foil was performed with plant-less pots, wood stakes, and metal paper clips (data not shown). 

#### 4.4.3. Chemicals 

α-pinene, camphene, β-pinene, D-limonene, *cis*-β-ocimene, *trans*-β-ocimene, γ-terpinene, α-terpinolene, (*Z*)-3-hexenylacetate, (*E*)-3-hexenylacetate, linalool, methyl salicylate, longicyclene, β-caryophyllene, α-humulene, valencene, (*E-E*)-α-farnesene, and α-sabinene were purchased from Merck (Isle d’Abeau Chesnes, Saint Quentin Fallavier, France). Stock solutions of each standard were prepared at 10 g/L in ethanol (ethanol absolute, VWR, Fontenay sous Bois, France) and kept at 4 °C. A stock solution of a mixture of all standards was prepared at 100 mg/L in ethanol and stored at 4 °C up to 6 months. For analysis, the stock solution was diluted successively to 1 mg/L in water (LC-MS grade water, Fisher Scientific, Illkirch, France) and then to 50 µg/L. An external calibration was performed with monoterpenes, sesquiterpenes, and green leaf volatile standards prepared in water in the 12.5 μg/L to 1000 μg/L concentration range. The final standard mixture solution was placed into a 20-mL headspace vial, and a stir bar was exposed in the headspace of the vial for 1 h at 50 °C. The VOCs collected on the stir bar were desorbed and analysed according to the method described below. Standards and parameters are listed in Supplemental Table S2.

#### 4.4.4. VOC Analysis by GC-MS

Analyses were carried on an Agilent 7890 Chromatograph hyphenated to a triple quadrupole mass spectrometer (7000C, Agilent Technologies, Les Ulis, France) equipped with an MPS robotic, thermal desorption unit (TDU 2) and a programmed temperature vaporiser inlet (PTV) from Gerstel (Gerstel GmbH, Co.KG, Mülheim an der Ruhr, Germany). After VOC sampling, the stir bars were kept at room temperature in their storage vials until they were placed in the thermal desorption unit (Gerstel, TDU 2). The stir bars were desorbed in splitless mode under a 50 mL/min helium flow at 270 °C for 3 min. Analytes were cryogenically trapped in the CIS 4 (Gerstel) inlet at 150 °C, on a baffled liner. Once the desorption step was completed, analytes were transferred on a 30-m length, 0.250-mm internal diameter, 0.25-µm film thickness GC column, (RTX-5MS Restek, Bellefontaine, PA, USA) in splitless mode by heating the CIS rapidly to 270 °C, with a hold time of 2 min. Analyses were performed under a constant helium flow of 1 mL/min. The GC oven temperature was held at 30 °C, then ramped up to 250 °C at a rate of 5 °C/min. The transfer line temperature was maintained at 280 °C. The analytes were ionised with an electron ionisation source at 70 eV maintained at 230 °C. Data were acquired on the MS1 quadrupole held at 150 °C using the scan mode. 

#### 4.4.5. GC-MS Data Processing and Statistical Analysis 

The acquired data were integrated and deconvoluted with Unknowns Analysis software, version B.08.00 (Agilent). The peaks detected after deconvolution were identified against two libraries—the NIST 14 library and a home-made one. The retention times of the peaks were checked against those of a set of standards analysed under the same conditions. The peaks due to PDMS coating were removed. The peaks were aligned according to their retention times using RStudio software before statistical treatment. A principal component analysis (PCA) of the log2-transformed mean values (RStudio) was performed. Significant compounds were determined by ANOVA multivariate statistical analysis (*p* < 0.05) using Perseus software (https://maxquant.net/perseus/, accessed on 6 April 2021). In addition to significance criteria, the compounds emitted by the elicited plants with a fold-change (FC) > 1.5 compared with the H_2_O treatment were also retained when induction was observed in at least two of the three replicates. For the elicitor-VOC list comparisons, only the FC > 1.5 obtained at least twice for one criteria (one elicitor or one time-point) was used (Table 2). For commonly used elicitor-VOC determination (Venn diagram, Figure 3), only induced VOCs (FC > 1.5) observed twice per elicitor treatment were compared.

### 4.5. Analysis of Farnesene Synthase (VvFAR) Gene Expression

Total RNAs were extracted from leaf samples from three plants collected 1 and 4 dpt (on D1 and D4). Samples were ground in liquid nitrogen, and extraction was performed with PureLink Plant RNA Reagent Extraction Buffer (Invitrogen, Thermofisher, Carlsbad, CA, USA) according to the manufacturer’s instructions. In order to eliminate residual genomic DNA, the samples were treated with a DNA-free DNAse removal kit (Invitrogen, Thermofisher, Carlsbad, CA, USA). Complementary DNA was synthesized from total RNAs with a Superscript III Reverse Transcriptase kit (Invitrogen™, Life Technologies, Saint Aubin, France). Real-time quantitative PCR was performed using an ABsolute SYBRGreen Low ROX qPCR Mix Kit (Thermo Scientific, Waltham, MA, USA) with an Applied Biosystems^®^ ViiA 7 apparatus (Life Technologies, Inc., Applied Biosystems, Foster City, CA, USA). The wells were loaded with the Tecan Freedom EVO 8 capillary robot on 384-well plates. Relative expression of the genes, with EF1-Ɣ and 60S as internal reference genes, was determined using the 2^−ΔΔCt^ method [51], with the water-treated sample as the negative control. Primers of the *VvFAR* gene coding for the (*E,E*)-α-farnesene synthase family (NCBI entry: GSVIVT01006399001 XM_02264098.4) were used, with the following sequences: AATATGGAGATGGGCTTGGA (forward primer) and CATGGACTTCCCAAGTACCC (reverse primer).

### 4.6. Stilbene Analysis

Leaves were harvested at five times points from D1 to D8 (Figure 6A). They were ground in liquid nitrogen to a fine powder that was freeze-dried, and 100 mg were extracted with 8 mL of methanol overnight at 4 °C. Six mL of supernatant were collected, evaporated, and the pellet was dissolved in 1 mL of methanol/water (70/30, *v/v*). The leaf extracts were purified to remove chlorophylls on a Supelclean LC-18 solid phase extraction column (Supelco Inc., Bellefonte, PA, USA). Eluates obtained with methanol/water (90/10, *v/v*) were evaporated and resuspended in 600 µL of methanol/water (50/50, *v/v*). The phenolic extracts were diluted, centrifuged at 13,500 rpm for 10 min, and stored at −20 °C until use. 

Stilbenes were analysed by a multiple reaction monitoring (MRM) mode HPLC-MS/MS methodology adapted from Krzyzaniak, et al. [52]. The device consisted of a 1260 Infinity UPLC system (Agilent Technologies, Courtaboeuf, France) coupled to a 6430 triple quadrupole mass spectrometer (Agilent Technologies, Les Ulis, France). Four microlitres of leaf extract were injected into an Agilent Poroshell 120 EC-C18 column (150 mm × 2.1 mm, 2.7 µm diameter) thermostated at 30 °C. A flow rate of 0.3 mL/min of solvent A (99.9% distilled water, 0.1% formic acid) and solvent B (99.9% acetonitrile, 0.1% formic acid) was used with the following gradient: 0–5 min, 5–18% B; 5–15 min, 18–43% B; 15–17 min, 43–95% B; 17–20 min, 95% B; 20–22 min, 95–5% B. Acquisitions were done in the negative mode with the following source parameters: gas temperature 350 °C, voltage 3000 V, gas flow 1 L/min, nebuliser pressure 15 psi. Calibration curves (concentrations ranging from 0.004 to 10 mg/L) of pure standards (*trans*-resveratrol and *trans*-piceid, 3 replicates) were established to determine the stilbene concentrations in mg/g dry weight (DW). Both standard stilbenes were produced and purified in laboratory conditions (Oenology Research unit, Bordeaux university, Villenave d’Ornon, France). *cis*-resveratrol and *cis*-piceid were expressed as their corresponding *trans*-isomers. Supplemental Table S3 describes the detection method and quantification parameters.

## Figures and Tables

**Table 1:** Bastid^®^-induced VOCs: VOCs analysed by SBSE-GC-MS were selected when significant (*p* < 0.05) and induced (FC > 1.5) at least twice in three separate experiments, compared to the water control. Coloured boxes, Bastid^®^ / H_2_O induction ratios 1, 3, and 5 days post treatment (on D1, D3 and D5), with ■ fold change (FC) < 1.5 (not induced); ■ 1.5–10^2^; ■ 10^2^–10^3^; ■ 10^3^–10^4^; ■ 10^4^–10^5^; ■ > 10^5^, □ not detected. Fold changes and day discrimination significance were determined from the three replicated experiments and averaged (right columns). The frequencies of ratios FC > 1.5 were summed for each day post-treatment (D1, D3, and D5) from the three replicated experiments and represented as follows: none, never detected; ◯ = 1, detected in one experiment with FC > 1.5 on the corresponding day post treatment; ● = 2, detected in two experiments with FC > 1.5 on the corresponding day post treatment; **●** = 3, detected in all three experiments with FC > 1.5 on the corresponding day post treatment. The CAS number (CAS **#**) of each VOC is indicated.

			Experiment 1				Experiment 2				Experiment 3				Mean ÷ Value		
Compounds	CAS #	Formula	D1	D3	D5	*p*	D1	D3	D5	*p*	D1	D3	D5	*p*	D1	D3	D5
α-Farnesene	21499-64-9	C15H24				0.0025								<0.001	**●**	**●**	**●**
β-Caryophyllene	87-44-5	C15H24								<0.001						**●**	**●**
2-Oxo-4-phenyl-6-(4-chlorophenyl)-1,2-dihydropyrimidine	24030-13-5	C16H11ClN2O				0.0233								0.0338	●	●	**●**
4,4’-bi-4H-pyran, 2,2’,6,6’-tetrakis(1,1-dimethylethyl)-4,4’-dimethyl-	1000399-10-0	C28H46O2				0.0346									●	**●**	
Toluene	108-88-3	C7H8				0.0430											●
3,4-Dihydroisoquinolin-7-ol, 1-[4-hydroxybenzyl]-6-methoxy-	47145-46-0	C17H17NO3				0.0018									●		**●**
Decanal	112-31-2	C10H20O				0.0509									●	**●**	**●**

**Table 2:** VOCs induced by the commercial elicitors Bastid^®^, Redeli^®^, and Romeo ^®^. List of the VOCs induced (FC > 1.5 compared to the water control) at least twice by Bastid^®^, Redeli^®^ or Romeo ^®^ treatment (three replicates/treatment). Coloured boxes indicate average elicitor/H_2_O induction ratios 1, 3, and 5 days post treatment (D1, D3, and D5), with ■ fold change (FC) < 1.5 (not induced); ■ 1.5–10^2^; ■ 10^2^–10^3^; ■ 10^3^–10^4^; ■ 10^4^–10^5^; ■ > 10^5^, □ not detected. For each elicitor, fold changes were determined from the three replicates and averaged. For each elicitor, the frequencies of the FC > 1.5 ratios were summed from the three replicates for each day post-treatment (D1, D3 and D5), and represented as follows: none = never detected; ◯ = 1, detected in one replicate with FC > 1.5 on the corresponding day post treatment; ● = 2, detected in two replicates with FC > 1.5 on the corresponding day post treatment; and **●** = 3, detected in three replicates with FC > 1.5 on the corresponding day post treatment. Bold type, VOCs significantly induced by Bastid^®^ (Table 1). The CAS number (CAS **#**) of each VOC is indicated. “Group” indicates the elicitor(s) when the VOC fitted at least two ◯ and/or one ●.

			Bastid^®^			Redeli^®^			Romeo^®^			
Compounds	CAS #	Formula	D1	D3	D5	D1	D3	D5	D1	D3	D5	Group
**α-Farnesene**	21499-64-9	**C15H24**	●	●	●		●		●	●	●	all
**2-Oxo-4-phenyl-6-(4-chlorophenyl)-1,2-dihydropyrimidine**	24030-13-5	C16H11ClN2O	●	●	●	●	●		●	●		all
Oxime-, methoxy-phenyl-	1000222-86-6	C8H9NO2	●	●	●	●			●		●	all
**4,4’-bi-4H-pyran, 2,2’,6,6’-tetrakis(1,1-dimethylethyl)-4,4’-dimethyl-**	1000399-10-0	C28H46O2	●	●		●		●	●	●	●	all
1,3-benzenedicarboxylic acid, 5-(dimethylamino)-	1000400-59-0	C10H11NO4		●	●	●	●	●		●	●	all
**β-Caryophyllene**	87-44-5	**C15H24**		●	●		●	●		●	●	all
Tridecane	629-50-5	C13H28	●	●	●	●		●	●	●		all
Decane, 3,8-dimethyl-	17312-55-9	C12H26		●	●	●	●	●		●	●	all
Cyclobutanol	2919-23-5	C4H8O		●	●	●	●		●	●	●	all
Decane, 2,3,8-trimethyl-	62238-14-6	C13H28		●		●	●	●	●	●	●	Redeli/Romeo
**Decanal**	112-31-2	C10H20O	●	●	●	●					●	Bastid
Dodecane	112-40-3	C12H26	●	●					●	●	●	Bastid/Romeo
**Toluene**	108-88-3	C7H8			●		●		●	●		all
Carbazole, 2,4,6-trimethyl-	78787-89-0	C15H15N	●	●		●		●		●		all
4-Hexen-1-ol-acetate	72237-36-6	C8H14O2		●	●	●	●			●	●	all
Limonene	5989-27-5	**C10H16**			●	●	●	●	●	●		Redeli/Romeo
Nonane, 2,2,4,4,6,8,8-heptamethyl-	4390-04-9	C16H34		●	●			●				Bastid
**3,4-Dihydroisoquinolin-7-ol, 1-[4-hydroxybenzyl]-6-methoxy-**	47145-46-0	C17H17NO3	●		●			●			●	Bastid
n-Hexadecanoic acid	112-39-0	C16H32O2	●		●		●				●	Bastid
Benzene, (1-ethyldecyl)-	2400-00-2	C18H30	●	●	●				●		●	Bastid/Romeo
Borane, diethyl(decyloxy)-	1000152-34-3	C14H31BO		●	●	●	●			●		Bastid/Redeli
Undecane, 3,5-dimethyl-	17312-82-2	C13H28		●	●			●		●		Bastid/Romeo
Sulphurous acid, 2-ethylhexyl hexyl ester	1000309-20-2	C14H30O3S	●		●	●			●			Bastid/Romeo
Succinic acid, 2,4,6-trichlorophenyl 2-naphthylmethyl ester	1000390-01-0	C21H15Cl3O4				●	●		●	●	●	Redeli/Romeo
2,5-Cyclohexadiene, 1,4-diethyl-1,4-dimethyl-	1000150-21-6	C12H20		●	●					●		Bastid
3-Pentanamine	616-24-0	C5H13N		●				●			●	Bastid
Benzene, (1-butyloctyl)-	2719-63-3	C18H30	●	●					●		●	Bastid/Romeo
Pyrimidine-2,4(1H,3H)-dione, 5-amino-6-nitroso-	1000270-67-7	C4H4N4O3			●	●	●	●				Redeli
3-Ethyl-3-methylheptane	17302-01-1	C10H22			●	●		●		●		Redeli
1,2-Benzenedicarboxylic acid, bis(2-methylpropyl) ester	84-69-5	C16H22O4	●			●				●	●	Romeo
4-Chlorobenzoic acid, 4-nitrophenyl ester	1000307-76-2	C13H8ClNO4				●	●		●	●		Redeli/Romeo
Benzaldehyde	78725-46-9	C7H6O					●	●		●	●	Redeli/Romeo
Methenamine	100-97-0	C6H12N4				●			●	●	●	Romeo
α-Pinene	80-56-8	C10H16	●		●			●				Bastid
Nonanal	124-19-6	C9H18O	●								●	Bastid
Methyl methacrylate	80-62-6	C5H8O2		●	●						●	Bastid
*cis*-muurola-4(14),5-diene	157477-72-0	C15H24	●					●				Redeli
3-Benzoyl-2-t-butyl-4-isopropyloxazolidin-5-one	104057-68-3	C17H23NO3				●		●			●	Redeli
Undecane, 2,6-dimethyl-	17301-23-4	C13H28				●		●		●		Redeli
Methyl salicylate	119-36-8	C8H8O3						●		●	●	Romeo
Tetradecane	629-59-4	C14H30				●			●		●	Romeo
Butanoic acid, 2-methyl-, 1,2-dimethylpropyl ester	84696-83-3	C10H20O2					●					Redeli
4-Ethylbenzoic acid, 2-formyl-4,6-dichlorophenyl ester	1000331-31-6	C16H12Cl2O3							●	●		Romeo

**Table 3:** Elicitors and doses used in the study. DP: degree of polymerisation.

Product	Manufacturer	Active Component	Doses
Bastid^®^	Syngenta (Guyancourt, France)	chitooligosaccharide-oligogalacturonic acid	0.8 mL/L
chitosan	Elicityl (Crolles, France)	chitooligosaccharide DP ~13	1 g/L
OG	Goëmar (Saint-Malo, France)	oligogalacturonic acid DP ~50	2.5 g/L
Romeo^®^	BASF (Ecully, France)	yeast cell wall (*Saccharomyces cerevisiae* LAS117)	2 g/L
Redeli^®^	Syngenta (Guyancourt, France)	disodium phosphonate	1 g/L
Bion^®^ WG 50	Syngenta (Guyancourt, France)	acibenzolar-S-methyl	0.5 g/L

## Data Availability

Analyzed data are provided in the table of the article.

## Supplementary Materials

The following are available online. Figure S1: Time-dependent VOC emission. Table S1: VOCs induced by Bastid^®^, chitosan, an oligogalacturonide (OG), and Bion^®^. Table S2: Parameters of the VOC calibration standards. Table S3: Parameters of the stilbene analysis method.

## Sample Availability

No available samples.
