# Supplementary material for: VOCs Are Relevant Biomarkers of Elicitor-Induced Defences in Grapevine
Source: Molecules. 2021 Jul 13;26(14):4258. doi: 10.3390/molecules26144258 (PMC8306312; doi:10.3390/molecules26144258)
Supplement: Supplementary file 1 [file molecules-26-04258-s001.zip › Molecules_CLemaitreGuillier_ Figure S1.pdf]

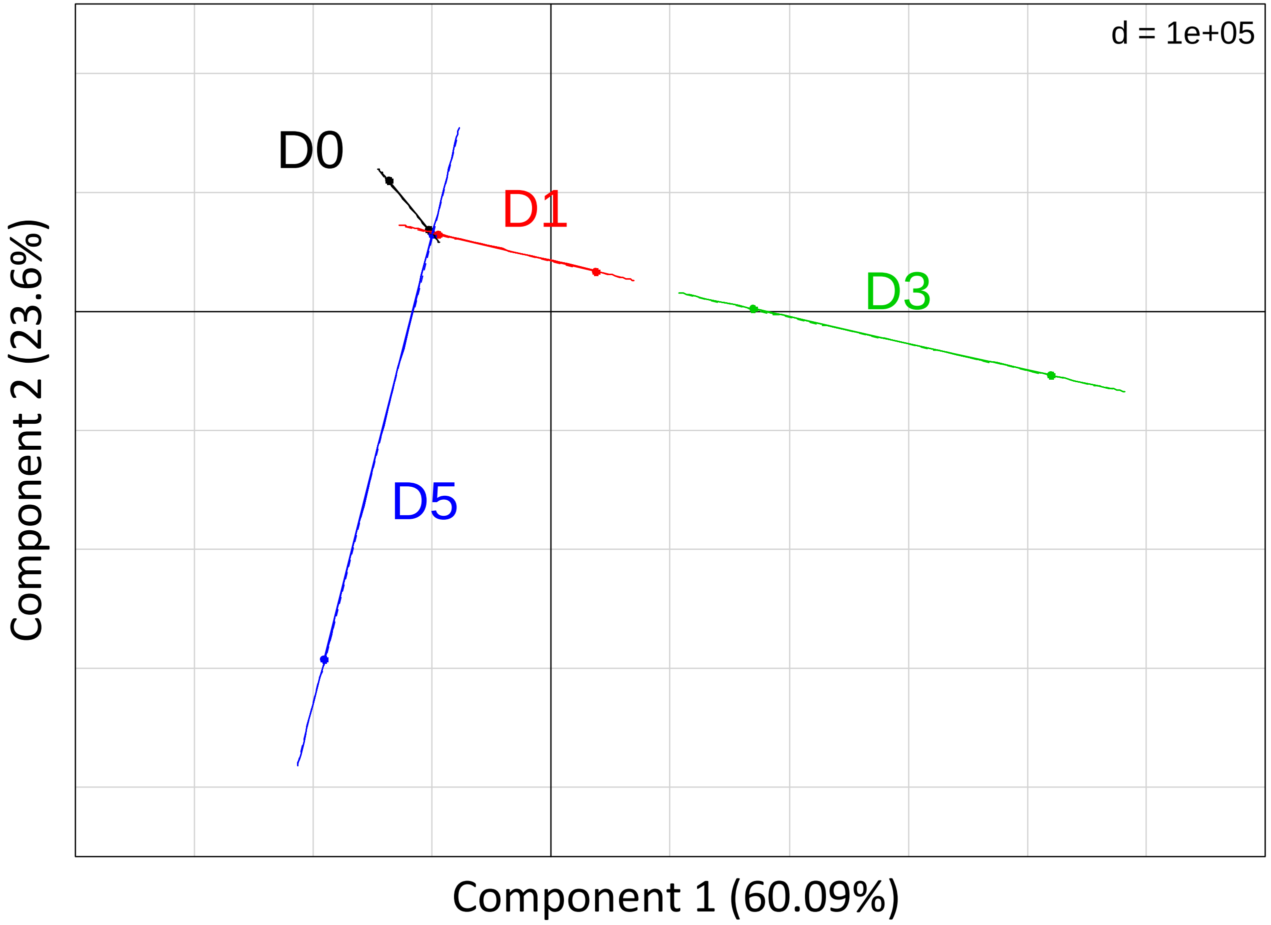


**Supplementary Figure S1. Time-dependent VOC emission.** Principal component analysis (PCA) illustration of a representative experiment performed on 6 Bastid-treated plants confined in tent. VOCs were collected (2 stir bars, 4 hours) at D0 (no treatment), D1, D3 and D5 after treatment. PCA analysis was performed by RStudio (vegan package) on 186 compounds.
