# Supplementary material for: VOCs Are Relevant Biomarkers of Elicitor-Induced Defences in Grapevine
Source: Molecules. 2021 Jul 13;26(14):4258. doi: 10.3390/molecules26144258 (PMC8306312; doi:10.3390/molecules26144258)

Supplementary Table S1. VOCs induced by Bastid®, chitosan, an oligogalacturonide (OG), and Bion®: List of VOCs selected when induced (FC > 1.5) at least twice over the experiments repeats (Bastid® and OG: 3 repeats, chitosan: 2 repeats, Bion®: 1 repeat). Coloured boxes indicate elicitor / H_2O averaged ratio at 1-, 3- and 5-days post treatment (D1, D3 and D5): Fold change (FC) wide range: ■ : < 1.5 (not induced); ■ : 1.5 - 10^2; ■ : 10^2 - 10^3; : ■10^3 - 10^4 : ■ ;10^4 - 10^5 > : ■ ;10^5□ , : not detected. Fold changes and day discrimination significance were determined from the repeated experiments and averaged (right columns). Frequencies of ratios FC > 1.5 were summed for each day post-treatment (D1, D3 and D5) from the 3 separated experiments and symbolized as follows: none = never detected induced, ○ = 1, detected in one repeat with FC > 1.5 at the corresponding day post-treatment, ● = 2, detected in two repeats with FC > 1.5 at the corresponding day post-treatment and ● = 3, detected in three repeats with FC > 1.5 at the corresponding day post-treatment. Bolded listed VOCs are the significantly induced Bastid® ones (Table 1). CAS number (CAS #) of VOC is indicated.

Compounds	CAS #	Formula	Bastid®			chitosan			OG			Bion®		
			D1	D3	D5	D1	D3	D5	D1	D3	D5	D1	D3	D5
**α-Farnesene**	21499-64-9	C15H24	○	●	●			○		◍			○	○
**β-Caryophyllene**	87-44-5	C15H24		○	○		○			◍	◍		○	
Oxime-, methoxy-phenyl-_	1000222-86-6	C8H9NO2	◍	●	○				◍	○	○			
**2-Oxo-4-phenyl-6-(4-chlorophenyl)-1,2-dihydropyrimidine**	24030-13-5	C16H11ClN2O	◍	◍	○	○			○	●	○			
**4,4'-bi-4H-pyran, 2,2',6,6'-tetrakis(1,1-dimethylethyl)-4,4'-dimethyl-**	1000399-10-0	C28H46O2	◍	○		○					○			
**Toluene**	108-88-3	C7H8			◍			○	○	◍	○		○	
cis-α-Bergamotene	13474-59-4	C15H24		○									○	
Decane, 3,8-dimethyl-	17312-55-9	C12H26		○	○				◍	○		○	○	
1,3-benzenedicarboxylic acid, 5-(dimethylamino)-	1000400-59-0	C10H11NO4		◍	○		○	○		○			○	
Tridecane	629-50-5	C13H28	○	○	○				○	◍			○	
**Decanal**	112-31-2	C10H20O	○	○	○	○	○	○					○	
Nonane, 2,2,4,4,6,8,8-heptamethyl-	4390-04-9	C16H34		○	○		○						○	
3-Pentanamine	616-24-0	C5H13N		◍		○						○		○
Carbazole, 2,4,6-trimethyl-	78787-89-0	C15H15N	○	○					○	◍			○	
Benzene, (1-ethyldecyl)-	2400-00-2	C18H30	○	○	○				◍	◍	◍			
n-Hexadecanoic acid	112-39-0	C16H32O2	◍		○				○	○			○	
Dodecane	112-40-3	C12H26	○	◍					○		○		○	
Nonanal	124-19-6	C9H18O	◍							○	○		○	
Undecane, 3,5-dimethyl-	17312-82-2	C13H28		○	○				○	○	○			○
Sulfurous acid, 2-ethylhexyl hexyl ester	1000309-20-2	C14H30O3S	○		○				○		○	○		
α-Pinene	80-56-8	C10H16	○		○								○	○

4-Hexen-1-ol-acetate	72237-36-6	$C_8H_{14}O_2$
Cyclobutanol	2919-23-5	C_4H_8O
Benzene, (1-butyloctyl)-	2719-63-3	$C_{18}H_{30}$
Borane, diethyl(decyloxy)-	1000152-34-3	$C_{14}H_{31}BO$
cis-muurola-4(14),5-diene	157477-72-0	$C_{15}H_{24}$
Decane, 2,3,8-trimethyl-	62238-14-6	$C_{13}H_{28}$
Sulfurous acid, butyl decyl ester	1000309-20-2	$C_{14}H_{30}O_3S$
1,2-Benzenedicarboxylic acid, bis(2-methylpropyl) ester	84-69-5	$C_{16}H_{22}O_4$
Benzene, (1-methylundecyl)-	2719-61-1	$C_{18}H_{30}$
1,3-Butadiene, 1,1,2,3,4,4-hexachloro-	87-68-3	C_4Cl_6
Benzene, (1-propyloctyl)-	4536-86-1	$C_{17}H_{28}$
Limonene	5989-27-5	$C_{10}H_{16}$
Benzene, 1,3-dimethyl-	108-38-3	C_8H_{10}
2,3,4,6-Tetrahydroisoquinolin-6-one, 1-[4-hydroxybenzyl]-7-methoxy-	128944-43-4	$C_{17}H_{17}NO_3$
Pyrimidine-2,4(1H,3H)-dione, 5-amino-6-nitroso-	1000270-67-7	$C_4H_4N_4O_3$
3-Ethyl-3-methylheptane	17302-01-1	$C_{10}H_{22}$
Acetic acid	64-19-7	$C_2H_4O_2$
Octadecanoic acid	57-11-4	$C_{18}H_{36}O_2$
N-Formylglycine	2491-15-8	$C_3H_5NO_3$
Triacontane	638-68-6	$C_{30}H_{62}$
Bicyclo[7.2.0]undec-4-ene, 4,11,11-trimethyl-8-methylene-,[1R-(1R*,4Z,9S*)]- (*cis*-Caryophyllene)	118-65-0	$C_{15}H_{24}$
β-ocimene	13877-91-3	$C_{10}H_{16}$
2-Propanone, 1-methoxy-	5878-19-3	$C_4H_8O_2$
1,2-Benzenediol, o-(4-butylbenzoyl)-o'-(2-methylbenzoyl)-	1000325-96-0	$C_{25}H_{24}O_4$
Piperazine-2,5-dione, 4-(4-methylbenzoyl)-	259228-04-1	$C_{12}H_{12}N_2O_3$
Tetracosane	646-31-1	$C_{24}H_{50}$
Methyl salicylate	119-36-8	$C_8H_8O_3$
4-Ethylbenzoic acid, 2-formyl-4,6-dichlorophenyl ester	1000331-31-6	$C_{16}H_{12}Cl_2O_3$
Acetophenone	98-86-2	C_8H_8O
Naphthalene, 2-methyl-	91-57-6	$C_{11}H_{10}$
4-Undecene, 4-methyl-	61142-40-3	$C_{12}H_{24}$
Benzaldehyde	78725-46-9	C_7H_6O
4-Chlorobenzoic acid, 4-nitrophenyl ester	6264-29-5	$C_{13}H_8ClNO_4$

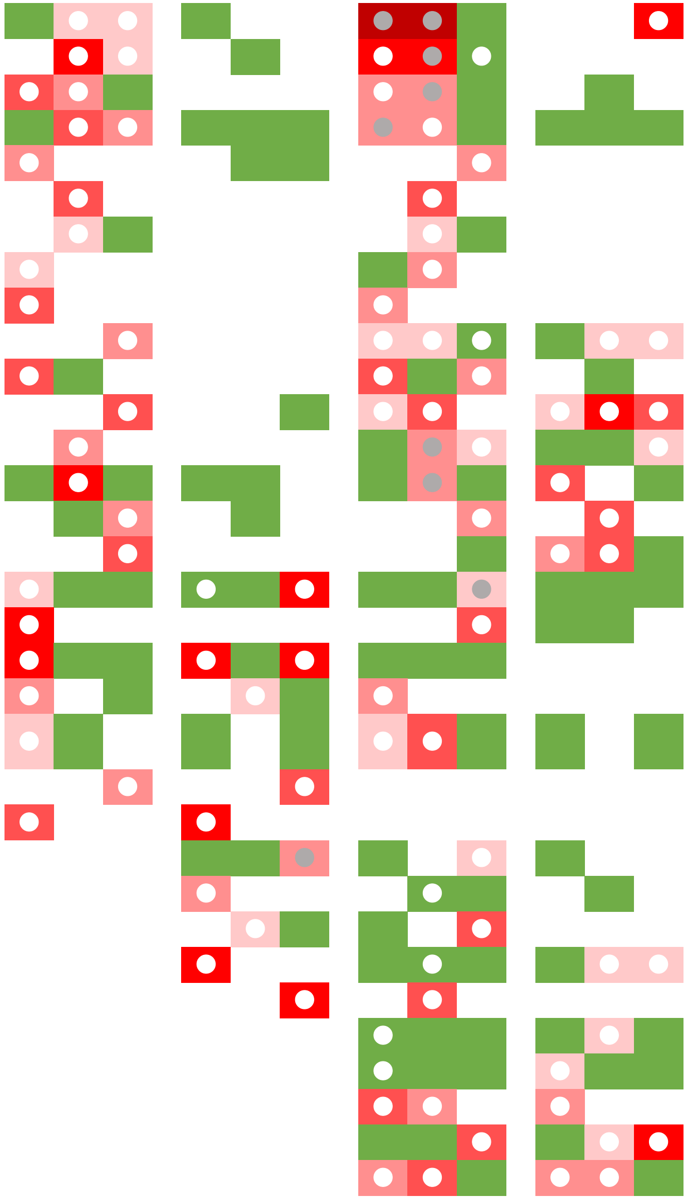

3-Benzoyl-2-t-butyl-4-isopropyloxazolidin-5-one	104057-68-3	C17H23NO3
Benzene, 1-isocyano-3-methyl-	621-29-4	C8H7N
Undecane, 6,6-dimethyl-	17312-76-4	C13H28
Benzaldehyde, 2,4-dichloro-	874-42-0	C7H4Cl2O
Succinic acid, 2,4,6-trichlorophenyl 2-naphthylmethyl ester	1000390-01-0	C21H15Cl3O4
5-Hepten-2-one, 6-methyl-	110-93-0	C8H14O
Undecane, 2,6-dimethyl-	17301-23-4	C13H28
Methenamine	100-97-0	C6H12N4
Tetradecane	629-59-4	C14H30
Acenaphthene	83-32-9	C12H10
1,1,5-Trimethyl-1, 2-dihydronaphthalene	1000357-25-8	C13H16
Naphtho[2,1-b]furan	232-95-1	C12H8O
Urea, 2-propenyl-	557-11-9	C4H8N2O

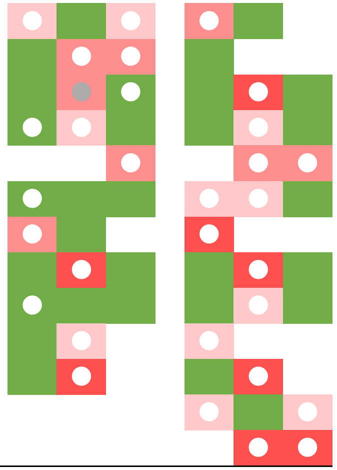

Supplement: Supplementary file 1 [file molecules-26-04258-s001.zip › Molecules_CLemaitreGuillier_Table S1.pdf]
