# Supplementary material for: VOCs Are Relevant Biomarkers of Elicitor-Induced Defences in Grapevine
Source: Molecules. 2021 Jul 13;26(14):4258. doi: 10.3390/molecules26144258 (PMC8306312; doi:10.3390/molecules26144258)
Supplement: Supplementary file 1 [file molecules-26-04258-s001.zip › Molecules_CLemaitreGuillier_Table S2.pdf]

Retention time	Compound name	CAS#	LOD (µg/L) S/N=3	LOQ (µg/L) S/N=10
8.77	α-Pinene	80-56-8	5.4	17.9
9.16	Camphene	79-92-5	4.5	14.8
9.90	α-Sabinene	3387-41-5	5.2	17.4
10.02	β-Pinene	127-91-3	2.5	8.5
11.06	3-Hexen-1-ol, acetate, (Z)-	3681-71-8	0.6	2.1
11.91	Limonene	138-86-3	2.0	6.7
12.32	*trans*-β-ocimene	3779-61-1	11.6	38.7
12.74	*cis*-β-Ocimene	3338-55-4	3.3	11.1
13.17	γ-Terpinene	99-85-4	1.8	6.1
14.45	α-Terpinolene	586-62-9	1.4	4.7
14.95	Linalool	78-70-6	6.8	22.6
18.99	Methyl salicylate	119-36-8	0.8	2.6
26.16	Longicyclene	1137-12-8	0.6	2.0
28.05	β-Caryophyllene	87-44-5	0.7	2.2
29.30	Humulene	6753-98-6	0.6	1.9
30.81	Valencene	4630-07-3	0.5	1.6
31.31	α-Farnesene	502-61-4	10.7	35.7

Supplementary Table S2. Parameters of the VOC calibration standards.
