# Supplementary material for: VOCs Are Relevant Biomarkers of Elicitor-Induced Defences in Grapevine
Source: Molecules. 2021 Jul 13;26(14):4258. doi: 10.3390/molecules26144258 (PMC8306312; doi:10.3390/molecules26144258)
Supplement: Supplementary file 1 [file molecules-26-04258-s001.zip › Molecules_CLemaitreGuillier_Table S3.pdf]

Compound	Retention time (min)	MRM	Calibration curve	Regression coefficient (R2)	Calibration Range (mg/L)	LOD (ug/g DW)	LOQ (ug/g DW)	Intraday variability (RSD%)	Interday variability (RSD%)
t-piceid	11.986	389→227	*Y= 73246x + 678*	0.9991	0.004-10	0.05	0.16	2.5%	13%
c-piceid	15.608	389→227	as *t-piceid*					1 .8%	5.0%
t-resveratrol	16.187	227→143	*Y=11536x+172*	0.99899	0.008-10	0.100	0.320	8.4%	17.7%
c-resveratrol	16.857	227→143	as *t-resveratrol*					8.7%	21%

Supplementary Table S3. Parameters of the stilbene analysis method. The lowest calibration point with a S/N >5 was considered as the LOD and the LOQ was calculated as 3.3LOD. Both LOD and LOQ have been recalculated as μg compound/g sample (DW). Intraday variability consisted on the relative standard deviation (RSD%) of a QC injected on the same day (n=3); interday variability consisted on the relative standard deviation (RSD%) of a QC injected on three consecutive days (n=3).
